# Selective inhibition of matrix metalloproteinase-2 in the multiple myeloma-bone microenvironment

**DOI:** 10.18632/oncotarget.18103

**Published:** 2017-05-23

**Authors:** Gemma Shay, Marilena Tauro, Fulvio Loiodice, Paolo Tortorella, Daniel M. Sullivan, Lori A. Hazlehurst, Conor C. Lynch

**Affiliations:** ^1^ Tumor Biology Department, H. Lee Moffitt Cancer Center and Research Institute, Tampa, FL, USA; ^2^ Department of Pharmacy and Pharmaceutical Sciences, Università degli Studi di Bari “A. Moro”, Bari, Italy; ^3^ Blood and Marrow Transplantation and Cellular Immunology Department, H. Lee Moffitt Cancer Center and Research Institute, Tampa, FL, USA; ^4^ Hematopoietic Malignancy and Transplantation Program, West Virginia University, Morgantown, WV, USA

**Keywords:** bone marrow microenvironment, multiple myeloma, skeletal malignancies, matrix metalloproteinases, bone targeted MMP inhibitors

## Abstract

Multiple myeloma is a plasma cell malignancy that homes aberrantly to bone causing extensive skeletal destruction. Despite the development of novel therapeutic agents that have significantly improved overall survival, multiple myeloma remains an incurable disease. Matrix metalloproteinase-2 (MMP-2) is associated with cancer and is significantly overexpressed in the bone marrow of myeloma patients. These data provide rationale for selectively inhibiting MMP-2 activity as a multiple myeloma treatment strategy. Given that MMP-2 is systemically expressed, we used novel “bone-seeking” bisphosphonate based MMP-2 specific inhibitors (BMMPIs) to target the skeletal tissue thereby circumventing potential off-target effects of MMP-2 inhibition outside the bone marrow-tumor microenvironment. Using *in vivo* models of multiple myeloma (5TGM1, U266), we examined the impact of MMP-2 inhibition on disease progression using BMMPIs. Our data demonstrate that BMMPIs can decrease multiple myeloma burden and protect against cancer-induced osteolysis. Additionally, we have shown that MMP-2 can be specifically inhibited in the multiple myeloma-bone microenvironment, underscoring the feasibility of developing targeted and tissue selective MMP inhibitors. Given the well-tolerated nature of bisphosphonates in humans, we anticipate that BMMPIs could be rapidly translated to the clinical setting for the treatment of multiple myeloma.

## INTRODUCTION

Multiple myeloma is characterized by the clonal expansion of malignant plasma cells within the bone marrow [1]. Over time, myeloma cells induce extensive osteolysis *via* the activation of bone resorbing osteoclasts [2]. Despite recent advances in treatment, it remains an incurable disease [3, 4]. The bone microenvironment is essential for the survival of myeloma cells, disease progression and drug resistance with many host cell types now known to play key roles including mesenchymal bone stromal cells (MSCs) and osteoclasts [4, 5]. Targeting the bone microenvironment therefore represents a logical therapeutic strategy for the treatment of the disease. To this end, bisphosphonates such as zoledronate can bind to the skeleton due to their pyrophosphate analog backbone and induce osteoclast apoptosis during resorption [6]. Inhibiting osteoclast mediated bone resorption limits the release of sequestered factors such as transforming growth factorβ (TGFβ) that drive multiple myeloma growth [7]. Bisphosphonates, can delay the time to the first skeletal related event (SRE) and increase overall survival in the setting of multiple myeloma [8]. Given the success of bisphosphonates in the clinic and other agents that modulate the bone microenvironment including denosumab (an inhibitor of the osteoclastogenic factor receptor activator of nuclear κB ligand-RANKL) there is strong rationale for the further development of therapeutics that limit tumor-bone interaction [9].

Our group and others have shown that matrix metalloproteinases (MMPs), a 23 member family of enzymes that control extracellular matrix (ECM) remodeling, are key regulators of cancer-bone interaction in skeletal malignancy [10]. This is not only due to extracellular matrix remodeling but also because of their ability to regulate the activity and availability of many cytokines and growth factors. In multiple myeloma, individual MMPs including, but not limited to, MMP-1, -2, -9, -13 and -14 either correlate with the aggressiveness of the disease or are mechanistically implicated in its progression [11–18]. For example, MMP-2 is highly expressed in bone marrow aspirates of multiple myeloma patients and the co-culture of myeloma cells with bone marrow stromal cells results in enhanced activation of the enzyme [14, 17]. Our group has previously shown that stromal MMP-2 is critical for the progression of bone metastatic breast cancer [19]. Taken together, these data provide rationale for the inhibition of MMP-2 as a potential therapeutic approach for the treatment of multiple myeloma.

Despite their clear association with the progression of solid and hematological malignancies, enthusiasm for MMPs as therapeutic targets for cancer treatment has been dampened by the failure of small molecule MMP inhibitors (MMPIs) in human clinical trials [20, 21]. The reasons why MMP inhibitors failed clinically are several fold, including dose-limiting side effects and lack of specificity for individual MMPs [22]. MMP translational research in the post-clinical trial era has been focused on delineating which MMPs specifically contribute to disease progression and on the generation of highly selective inhibitors that spare the activity of other MMP and metazincin family members [21]. MMP-2 is widely expressed in tissues throughout the body and therefore targeted inhibition of the enzyme for the treatment of multiple myeloma could potentially result in systemic toxicity. To combat this, we reasoned that specific targeting of an MMP-2 inhibitor to the skeletal tissue may circumvent potential dose limiting toxicities. Previously, bisphosphonates have been shown to have inherent MMP inhibitory profiles albeit at high concentrations [23]. Subsequent to their administration, bisphosphonates accumulate in the skeleton and are rapidly cleared from the plasma and other organs [24, 25]. Using a bisphosphonic foundation, we therefore developed a series of reagents that are highly selective for MMP-2 over other MMPs, including the closely related gelatinase MMP-9 [26, 27]. Structurally based on tiliduronate these novel inhibitors, inhibit MMP-2 in the nanomolar range while maintaining their ability to bind to hydroxyapatite [26, 28–30]. In the current study, we examined these bone seeking MMP inhibitors (BMMPIs) for their *in vivo* efficacy in the setting of multiple myeloma using independent clinically relevant models of the disease (5TGM1 and U266), and addressed as proof of principle whether individual MMP activity, in this case MMP-2, could be selectively inhibited in skeletal tissue.

## RESULTS

### MMP-2 expression and localization in human multiple myeloma

**Figure 1:**
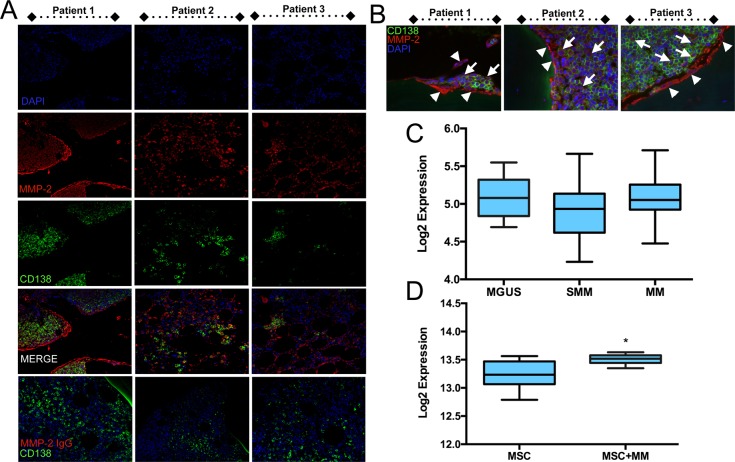
MMP-2 expression in human multiple myeloma biopsies **A**., MMP-2 (red) was detected by immunofluorescence in human biopsies of multiple myeloma (*n* = 10 with three representative patients illustrated). CD138 (green) was used to localize multiple myeloma cells in the biopsy specimens. DAPI (blue) was used as a nuclear counterstain. IgG control for MMP-2 staining is illustrated. Similar results were obtained for CD138 IgG control (data not shown). **B**., High magnification illustrates the presence of MMP-2 (red) in CD138 positive multiple myeloma cells (green, arrows) in addition to the surrounding stromal cells (arrow heads). IgG controls demonstrate the specificity of the MMP-2 antibody. **C**., Public database analysis (GEO accession GSE47552) of MMP-2 expression levels in CD138+ myeloma cells from monoclonal gammopathy of undetermined significance (MGUS, *n* = 20), smoldering multiple myeloma (SMM, *n* = 33) and multiple myeloma (MM, *n* = 41) specimens. **D**., Public database analysis (GEO accession GSE46053) of MMP-2 expression in normal human (*n* = 6) bone marrow mesenchymal stromal cells (MSCs) cultured either alone or in the presence MM.1S multiple myeloma (MM) cells for 24 hours using a transwell culture system. Asterisk denotes statistical significance with *p* < 0.05.

Given the possible roles for tumor and bone derived MMP-2 in driving the progression of skeletal malignancies we examined human multiple myeloma biopsies (*n* = 10) for the presence of MMP-2. Using immunofluorescence and CD138 to identify the myeloma cells, we observed that the majority of multiple myeloma cells in the bone marrow biopsies were positive for MMP-2 (Figure 1A and 1B). In keeping with our own studies and those from other groups, we found MMP-2 expression was widespread throughout the bone microenvironment [19]. Analysis of publically available datasets (GSE47552) examining gene expression in CD138+ isolated myeloma cells at various stages of disease progression did not demonstrate changes in MMP-2 (Figure 1C) [31]. However, analyzing differential gene expression in healthy human MSCs in response to co-culture with multiple myeloma cells (MM.1S) revealed a significant increase in MMP-2 expression (GSE46053 and Figure 1D) [32]. Based on the detection of MMP-2 in the myeloma-bone microenvironment and the role of the enzyme in driving cancer progression, we hypothesized MMP-2 inhibition may be a potential target for the treatment of the disease.

### The BMMPIs significantly limit osteoclast formation and survival *in vitro*

**Figure 2:**
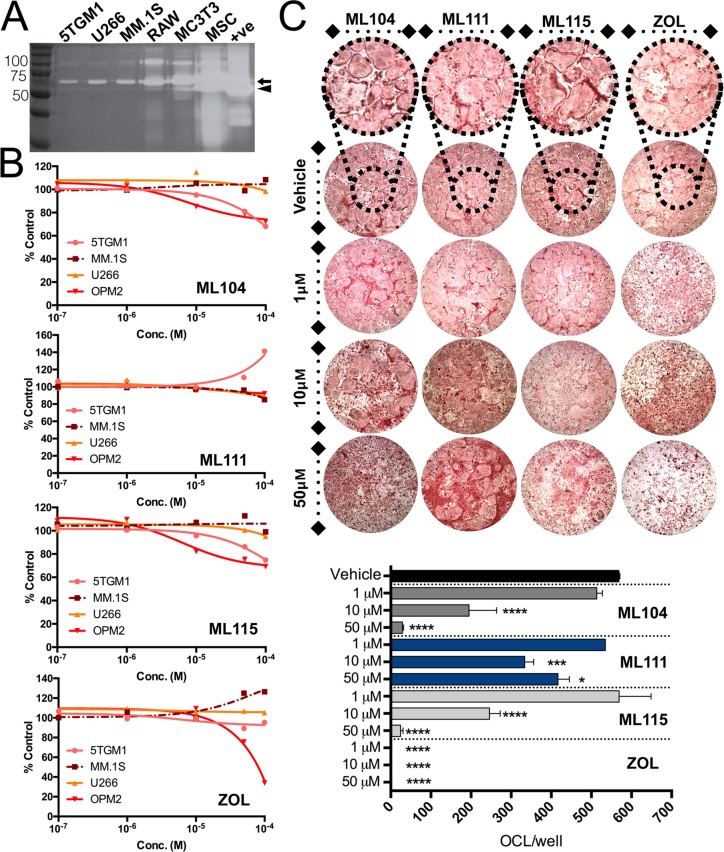
Impact of BMMPIs on multiple myeloma and osteoclast viability ***in vitro***. **A**., Analysis of MMP-2 expression in conditioned media derived from multiple myeloma cell lines (5TGM1, U266, MM.1S), the RAW 264.7 monocytic cell line (RAW) used for osteoclast differentiation, the osteoblast cell line, MC3T3-E1 and mesenchymal stromal cells (MSCs). Recombinant MMP-2 was used as a positive control (+ve). Molecular weight markers are shown in kiloDaltons (kDa). Arrow indicates latent MMP-2 at 72 kDa while arrow head indicates the active form of the enzyme at 66 kDa. **B**., Multiple myeloma cells lines (5TGM1, MM.1S, U266 and OPM2) were treated with varying concentrations of the BMMPIs, ML104, ML111 and ML115. The efficacy of the BMMPIs was compared to zoledronate (ZOL). Cells were incubated for 72 hours and viability was measured using an MTT assay. Results were normalized to a percentage of control. **C**., Osteoclast cultures were treated with the BMMPIs and zoledronate (ZOL) at varying concentrations for 24 hours. Inset of vehicle treated wells shows osteoclasts at higher magnification. The number of mature multinucleated osteoclasts/well of a 48 well plate (OCL/well) were counted. Asterisks denote statistical significance (*, *p* < 0.05, ***, *p* < 0.001, ****, *p* < 0.0001).

Bisphosphonates are commonly used for the treatment of multiple myeloma induced bone disease. While the primary mechanism of action of bisphosphonates is promoting osteoclast apoptosis, the reagents have also been shown to have inherent broad spectrum MMP inhibitory activity at high μM concentrations [23]. We therefore modified a bisphosphonate (tiludronate) structure to improve MMP-2 selectivity [27, 28, 33]. We hypothesized that the bisphosphonic nature of the inhibitors would ensure specific targeting to the skeleton, thereby avoiding potential dose limiting systemic toxicities noted in prior MMP inhibitor clinical trials [34]. As we reported, two BMMPIs, ML104 and ML115 had IC_50_s for MMP-2 in the nM range (37 and 140nM respectively) but importantly spare the activity of other MMPs including the closely related MMP-2 family member, MMP-9 [26, 35]. For these studies we also included another BMMPI, ML111 that did not show a high degree MMP-2 selectivity (IC_50_ = 4.9μM). Initially, we tested the impact of the inhibitors on the viability of the primary cellular components of the multiple myeloma-bone vicious cycle, namely the multiple myeloma cells (5TGM1, U266, OPM2 and MM.1S) and the osteoclast compartment. Each of the cell lines tested secrete MMP-2 at varying levels, but of note, the majority of the enzyme was in a latent state (Figure 2A). Results show that the highly selective MMP-2 based inhibitors (ML104 and ML115) impacted multiple myeloma cell line viability but only at higher concentrations (Figure 2B). Zoledronate and the non-MMP-2 selective BMMPI, ML111, also showed modest effects on multiple myeloma cell viability. We next examined the impact of the compounds on osteoclast viability. As expected, given their bisphosphonic nature, all of the BMMPIs including ML111 significantly limited osteoclast survival compared to vehicle controls although not as potently as zoledronate (Figure 2C). Our previous studies have also shown that BMMPIs do not affect osteoblast viability even at concentrations in excess of 100μM [35].

### Assessing the efficacy BMMPI compounds for multiple myeloma treatment *in vivo*

**Figure 3:**
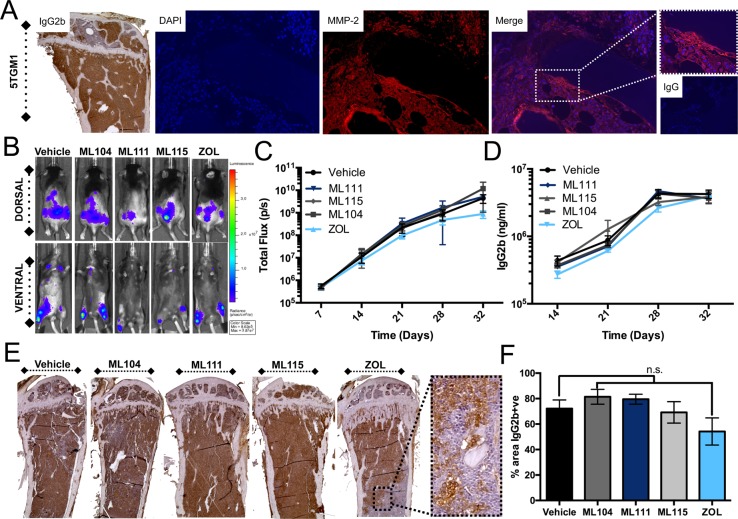
BMMPIs do not limit 5TGM1 growth ***in vivo***. **A**., Analysis of 5TGM1 bearing mice tibia (identified *via* IgG2b specific immunohistochemistry) demonstrates the expression of MMP-2 (red) in the multiple myeloma-bone microenvironment. IgG control for MMP-2 staining is illustrated was used as a negative control. DAPI (blue) was used a nuclear counterstain. 5TGM1 bearing mice were treated three times a week with vehicle, 1mg/kg BMMPIs (ML104, ML111 and ML115) or 100μg/kg ZOL. **B**., Whole body bioluminescence was measured over time in 5TGM1 bearing mice treated (*n* = 8 per group). Representative dorsal and ventral images are shown from the Day 21 time point. **C**., Quantitation of bioluminescence over time in each of the treatment groups. **D**., Analysis of serum levels of IgG2b in 5TGM1 tumor bearing mice over time. **E**. and **F**., Sections from tumor bearing tibias in each mice were stained for IgG2b **E**. Dashed box shows a representative high magnification image of IgG2b staining in zoledronate treated group. The tumor burden (% area of IgG2b positive staining in total area) was subsequently calculated using multiple tissue sections derived from animals in each group **F**. n.s. denotes that the p-value was non-significant.

While BMMPIs did not impact multiple myeloma cell line survival significantly *in vitro*, we expected that they may be effective in the context of the *in vivo* bone microenvironment given the combined presence of MMP-2 in the multiple myeloma and host compartments. To test this, 5TGM1 murine multiple myeloma cells expressing luciferase were inoculated (tail vein IV) into syngeneic immunocompetent C57BL/KaLwRij mice [36]. The 5TGM1 cells express MMP-2 *in vivo* as noted by immunofluorescent studies (Figure 3A). After 1 week, bioluminescence could be detected in the skeleton. Mice were subsequently randomized into vehicle, ML104, ML111, ML115, and zoledronate groups (*n* = 8 group) and treated as described. Using total bioluminescence as a readout for multiple myeloma growth over time we observed no significant differences in growth rates between the control and treatment arms (Figure 3B and 3C). This observation was supported by ELISA analysis of serum levels of 5TGM1 derived IgG2b over time (Figure 3D) and by quantitating tumor volume (IgG2b immunohistochemistry) as a function of total volume in tibia sections derived from animals in each group (Figure 3E and 3F). The clinical endpoint for the 5TGM1 model is typically hind limb paralysis. The median survival time for control treated animals was 31 days compared to ML104 (35 days), ML111 (30 days), ML115 (32.5 days), and, zoledronate (33 days). Despite the average median survival over control being 12.9% higher for ML104, this increase did not prove to be statistically significant. Animals tolerated BMMPIs well with no weight loss observed during the study and no remarkable gross differences in the appearance or size of isolated visceral organs (data not shown).

**Figure 4:**
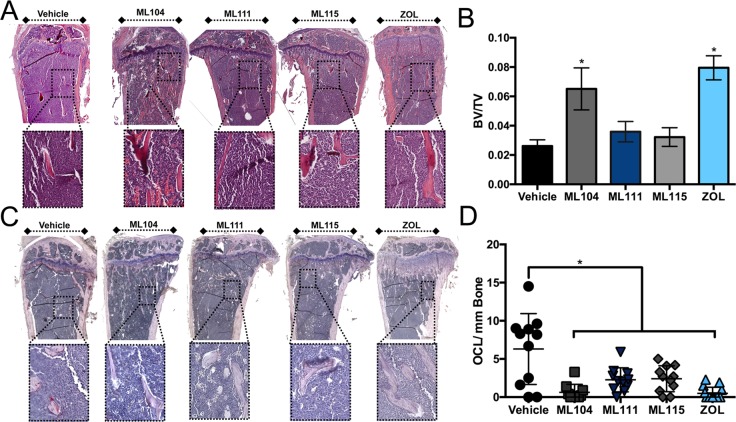
The BMMPI, ML104, protects against 5TGM1 induced bone loss **A**. and **B**., The trabecular bone volume (BV) in H&E stained tissue sections derived from tumor bearing tibias of mice in each group (A) was measured as a function of the total volume (TV, B). Representative images from each group are shown with dashed boxed defining an area of high magnification. The analyzed region of interest was comparable between treatment groups. **C**. and **D**., Osteoclast number was determined by TRAcP staining for osteoclasts. Representative images (C) illustrate areas of osteoclasts, primarily in the vehicle control group. Dashed box indicates area of high-magnification. The number of multi-nucleated TRAcP positive osteoclast per mm of bone was measured in tissue sections derived from tumor bearing tibias of mice in each vehicle and treatment group (D). Asterisks denote statistical significance (*, *p* < 0.05).

We next examined the impact of the inhibitors on bone disease. We observed that ML104 treated animals behaved similarly to zoledronate and had significantly more trabecular bone volume compared to vehicle control mice (Figure 4A and 4B). We also noted that BMMPI and zoledronate treated mice had significantly less osteoclast numbers than the vehicle control (Figure 4C and 4D). Taken together, our *in vivo* data demonstrate that the BMMPI, ML104 can significantly protect against multiple myeloma induced bone loss in the 5TGM1 model.

### The BMMPI ML104 inhibits multiple myeloma growth *in vivo* and associated bone loss

**Figure 5:**
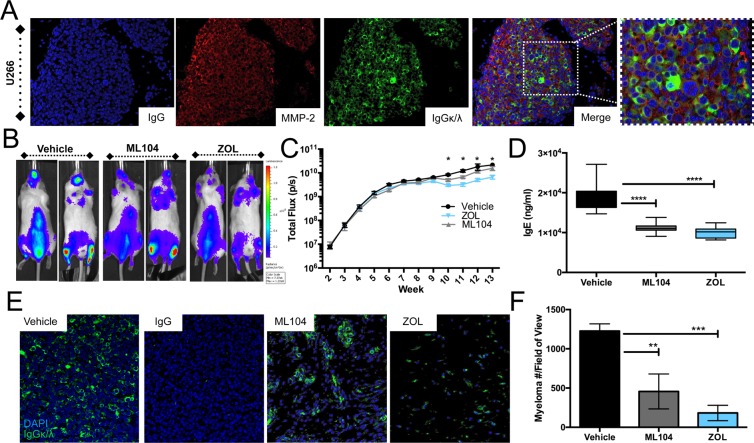
ML104 significantly impacts tumor burden in the U266 multiple myeloma model **A**., Analysis of U266 bearing mice tibia (identified by IgGκλ staining) demonstrates the expression of MMP-2 (red) in the multiple myeloma-bone microenvironment. DAPI (blue) was used a nuclear counterstain. IgG control for MMP-2 staining is illustrated. U266 bearing mice were treated three times a week with vehicle, 1mg/kg ML104 or 100μg/kg ZOL. **B**. and **C**., Whole body bioluminescence was measured over time in U266 bearing mice (*n* = 8 per group). Representative dorsal and ventral images are shown from the week 10 time point (B). Quantitation of total bioluminescence over time in each of the treatment groups presented on a logarithmic scale (C). **D**., Analysis of serum levels of IgE in U266 tumor bearing mice from week 10 presented on a linear scale. **E**. and **F**., Sections from tumor bearing tibias in each mice were stained for human Kappa and Lambda light chains (E, green) with DAPI (blue) used as a nuclear counter stain. The tumor burden (number of κ/λ positive myeloma cells/field of view) was subsequently calculated using multiple tissue sections derived from animals in each group (F). Asterisks denote statistical significance (*,*p* < 0.05, **, *p* < 0.01***, *p* < 0.001, ****, *p* < 0.0001).

Our data indicated that the BMMPI, ML104 was the superior BMMPI for preventing *in vivo* multiple myeloma induced bone disease. To validate the robustness of our *in vivo* studies, we examined whether ML104 would also be effective in a human model of multiple myeloma, U266. To this end, NOD-SCID mice were inoculated (tail vein IV) with luciferase expressing U266 cells. These cells home to the skeleton and induce osteolytic lesions over a 12-week period. U266 cells also express MMP-2 in the tumor-bone microenvironment as determined by immunofluorescence (Figure 5A). Upon detection of bioluminescence, mice were randomized into vehicle, ML104 and zoledronate groups (*n* = 8/group) and dosed accordingly. Using total bioluminescence as a readout for multiple myeloma growth, we observed that compared to vehicle control, both ML104 and zoledronate treated groups had a significantly lower tumor burden from week 10 onwards (Figure 5B and 5C). This difference was confirmed by analyses of serum levels of U266 derived IgE at week 10 and further by assessing myeloma tumor burden in isolated tibias (Figure 5D and 5E). Using clinical endpoints (hind limb paralysis, hunching and rapid weight loss), we observed that the median survival time for vehicle control treated animals was 81 days compared to ML104 (92 days) and, zoledronate (89.5 days). Despite the average median survival over control being 10-14% higher combined with significantly slower tumor growth rates, this increase in overall survival, similar to the 5TGM1 model, did not prove to be statistically significant.

**Figure 6:**
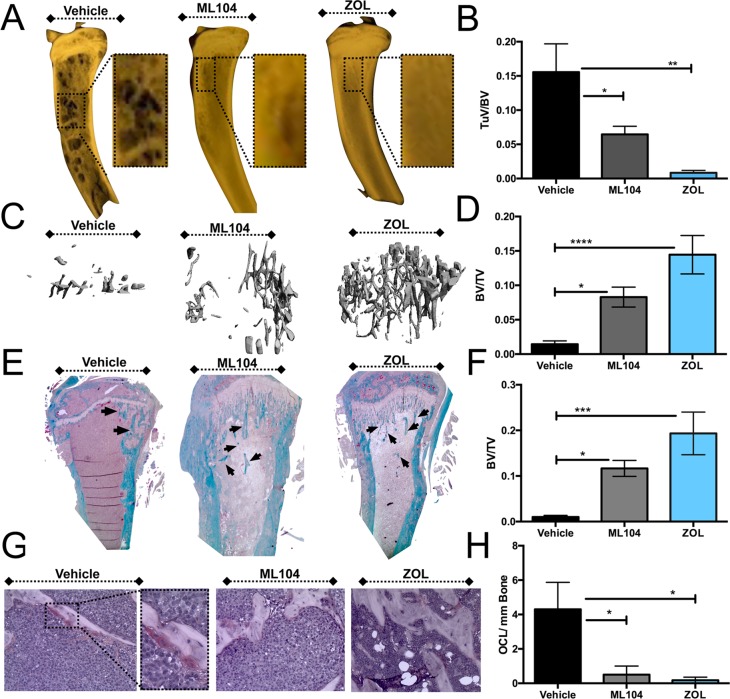
The BMMPI, ML104, protects against U266 induced bone loss **A**. and **B**., Representative images of Faxitron X-ray analyses from vehicle control, ML104 and ZOL treated U266 mice (A). Dashed box represents area of magnification. The volume of the osteolytic lesions (TuV) was calculated as a function of the total tibia volume from animals in each group (B, *n* = 4 mice/group). The analyzed region of interest was comparable between treatment groups. **C**. and **D**., Representative μCT scan analysis of the trabecular bone volume in each group (C). The bone volume (BV) was calculated as a function of total volume (TV). **E**. and **F**., The trabecular bone volume (BV, arrows) in trichrome stained tissue sections derived from tumor bearing tibias of mice in each group (E) was measured as a function of the total volume (TV, F). **G**. and **H**., Osteoclast number was determined by TRAcP staining for osteoclasts. Representative images (G) illustrate areas of osteoclasts, primarily in the vehicle control group. Inset defines a representative area of magnification. The number of multi-nucleated TRAcP positive osteoclast per mm of bone was measured in tissue sections derived from tumor bearing tibias of mice in each vehicle and treatment group (H). Asterisks denote statistical significance (*,*p* < 0.05, **, *p* < 0.01***, *p* < 0.001, ****, *p* < 0.0001).

Based on our observations with the 5TGM1 model, we were interested in further examination of the BMMPIs role in protecting against U266 myeloma induced bone loss. Isolated U266 tumor bearing limbs underwent X-ray imaging and analysis (Faxitron Ultrafocus). Osteolytic lesions could easily be identified in tumor bearing limbs derived from vehicle control treated animals compared to those derived from ML104 and zoledronate treated groups (Figure 6A). Measurement of osteolytic lesion size as a correlate for tumor volume supported bioluminescence and IgE analyses for the efficacy of ML104 and zoledronate in regards to limiting tumor burden (Figure 6B). High-resolution μCT analysis also demonstrated that ML104 and zoledronate significantly reduced the amount of multiple myeloma induced bone loss compared to vehicle controls (Figure 6C and 6D). Subsequent to *ex vivo* imaging, limbs were processed and sectioned. The protective effect of ML104 and zoledronate against myeloma induced bone disease was supported by histomorphometry analysis of trichrome stained sections (Figure 6E and 6F). In keeping with these observations, we also identified fewer TRAcP positive multinucleated osteoclasts in ML104 and zoledronate treated groups compared to control (Figure 6G and 6H). Collectively, these data confirm the beneficial effects of BMMPIs in protecting against multiple myeloma induced bone loss but also demonstrate their impact in effectively reducing tumor burden in a slower progressing model of the disease.

### BMMPIs selectively inhibit MMP-2 activity in the bone microenvironment

**Figure 7:**
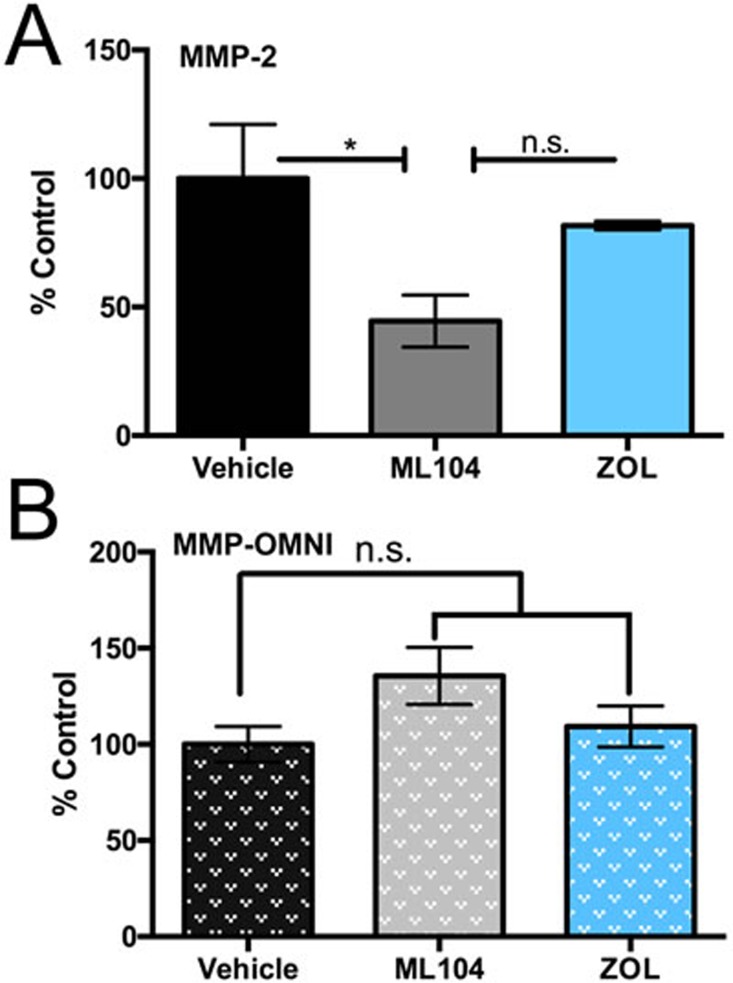
BMMPIs selectively inhibit MMP-2 activity in the bone marrow microenvironment **A**. and **B**., Bone marrow supernatants derived from U266 tumor bearing limbs of subsets of vehicle, ML104 and ZOL treated groups (*n* = 3/group dosed as described) at clinical endpoints were incubated with an MMP-2 specific (A), or broad spectrum MMP (OMNI, B) activatable fluorogenic peptides for 2 hour at 37°C. Asterisks denote statistical significance (*, *p* < 0.05) while n.s. denotes that the *p*-value was non-significant.

Our overarching goal in the current study was to determine whether MMP-2 activity could be selectively inhibited in the skeletal tissue by BMMPIs. To this end, we isolated whole bone marrow supernatants from the tibias of vehicle, ML104 and zoledronate treated multiple myeloma bearing animals (*n* = 3/group). Using an MMP-2 specific quenched fluorescent substrate, we demonstrated that ML104 significantly reduced MMP-2 activity in the multiple myeloma-bone microenvironment compared to either the control or zoledronate groups (Figure 7A). Similar studies using a broad spectrum MMP quenched fluorescent peptide showed no differences between the groups suggesting that ML104 was selectively inhibiting MMP-2 (Figure 7B). These data demonstrate the feasibility of selectively targeting MMP activity in bone for the treatment of multiple myeloma.

## DISCUSSION

The pivotal roles for MMPs in regulating tumor-bone interactions and bone matrix remodeling make them promising therapeutic targets for the treatment of skeletal malignancies [37]. Based on evidence that MMP-2 is expressed in the bone marrow of myeloma patients and known roles for MMP-2 in the progression of skeletal malignancies [11, 12, 14, 16, 19], we hypothesized that inhibiting MMP-2 in myeloma would be an effective therapeutic strategy to prevent disease progression. To address this, we created highly specific “bone-seeking” MMP-2 inhibitors (BMMPIs) to target the multiple myeloma-bone microenvironment. BMMPIs are structurally based on bisphosphonates ensuring that these agents target sites of multiple myeloma-induced osteolysis, while limiting the potential off-target effects of systemic MMP-2 inhibition. Our data demonstrate that BMMPIs significantly protect against multiple myeloma induced bone loss in a manner comparable to that of the standard of care, zoledronate. Importantly, we show selective inhibition of MMP-2 in the skeletal tissue is feasible.

Collected data also demonstrate the potential for BMMPIs reducing multiple myeloma burden. Data from our U266 studies show a significantly lower tumor burden in ML104 treated U266 mice compared to vehicle control. This is most likely due to the slower rate of progression in the U266 model (~10 weeks) compared to the more aggressive 5TGM1 model (~4 weeks). The U266 *in vivo* model has been proposed as a model of less aggressive myeloma, and early stage disease [38]. Proteomic analysis revealed that extracellular matrix remodeling may already take place during early asymptomatic stages of the disease with the up-regulation of extracellular remodeling proteins including MMP-2 noted [39]. This suggests that early application of ML104 would be most effective in myeloma treatment by limiting disease progression.

We also noted that the BMMPI, ML104, behaves similarly to zoledronate in protecting against multiple myeloma induced bone disease. On the surface, this would indicate that the efficacy of ML104 is due to the bisphosphonic nature of the reagent in promoting osteoclast apoptosis [6]. However, ML104 was significantly less potent at inhibiting osteoclast survival compared to zoledronate (Figure 2C) suggesting that inhibiting MMP-2 activity also contributes to the noted efficacy of the compound. Further, *ex vivo* analysis of bone marrow from multiple myeloma-bearing animals demonstrate the selective inhibition of MMP-2 activity in the bone microenvironment in the ML104, compared to zoledronate treated animals.

Given the contribution of MMP-2 to the progression of several skeletal malignancies, we posit that the combined MMP-2 inhibition and anti-osteoclast activity of ML104 explains why ML104 and zoledronate behave similarly *in vivo* in preventing multiple myeloma progression. While we demonstrate that MMP-2 is expressed in the multiple myeloma-bone microenvironment of human specimens (Figure 1), public database analyses did not reveal a correlation between MMP-2 and multiple myeloma stage at the gene expression level in CD138 isolated cells (Figure 1C). However, these analyses may not reflect the activity status of the enzyme. MMP-2 is a 72 kDa secreted proteinase that requires proteolytic cleavage of the pro-domain to yield an active 66 kDa form [40]. Analysis of conditioned media from multiple myeloma cell lines show that MMP-2 is expressed at the protein level but the majority of the enzyme is in a latent state (Figure 2A). This may explain why the BMMPIs do not impact the viability of the multiple myeloma cell lines *in vitro*. Previous reports have shown that MMP-2 expression is increased upon co-culture with stromal cells and our analysis of GEO46053 confirm this (Figure 1C) [32]. Studies also show that latent MMP-2 can be activated in the co-cultures of multiple myeloma and bone marrow stromal cells [16]. We would therefore predict that MMP-2 in the *in vivo* multiple myeloma-bone microenvironment is in an active state, which is supported by our *ex vivo* MMP-2 activity assays using bone marrow supernatants isolated from myeloma-bearing mice. These quantitative assays revealed a significant decrease in MMP-2 activity in ML104 treated mice compared to vehicle control and zoledronate. We posit that the reduced MMP-2 activity in combination with reduced osteoclast activation limits bone matrix remodeling and the availability of growth factors and cytokines that can drive multiple myeloma growth.

Previously, MMP inhibitors have been used in pre-clinical animal models of multiple myeloma. For example, SC-964 (a broad spectrum inhibitor of MMP-2, -3, 8, -9 and -13) significantly reduced 5TMM mouse multiple myeloma burden, osteolytic lesions and angiogenesis. These results further support the role of MMPs in controlling the progression of the disease [11]. In human clinical trials, broad-spectrum MMP inhibitors failed to reach their clinical endpoints for several reasons including dose-limiting toxicities such as tendonitis and arthralgia [41, 42]. Based on our studies, we show that specific targeting of individual MMPs in the multiple myeloma-bone microenvironment is feasible. We have shown that the BMMPIs are well tolerated with no side effects noted, even at doses of 25mg/kg, three times per week over the course of 4 weeks [35]. While the inhibition of MMP-2 had beneficial effects in reducing tumor burden and protecting against myeloma induced bone disease, targeting other individual MMPs known to play a role in multiple myeloma progression may be of benefit. We believe that with further refinement of the BMMPI structure and pharmodynamic studies, this therapeutic strategy could be more efficacious for the treatment of myeloma than the current standard of care bisphosphonates, such as zoledronate. For example, MMP-9 has been shown to control angiogenesis in multiple myeloma and genetic ablation of MMP-9 in an animal model can significantly delay the progression of the disease [13, 43, 44]. MMP-9 is closely related to MMP-2 and therefore modifying the BMMPI structure to target both of these gelatinases could have a more potent effect. We would expect that selective targeting of the skeletal tissue would again circumvent any potential off-target systemic effects. Currently, several therapeutic strategies are available for the treatment of multiple myeloma depending on the disease stage, with the majority of patients receiving bisphosphonates [45]. Our next step will be to determine the efficacy of BMMPIs in combination with clinically available multiple myeloma treatments such as, bortezomib. Bortezomib significantly reduces the expression of the type I collagenase MMP-13 and a key regulator of osteoclast fusion [15, 46]. Combination of BMMPIs that target other MMP family members expressed in the bone microenvironment such as MMP-2 and MMP-9 therefore may act synergistically with bortezomib for the treatment of multiple myeloma.

In conclusion, MMPs play key roles in the progression of skeletal malignancies such as multiple myeloma. Due to the failure of systemic administered broad-spectrum MMP inhibitors in the clinical setting, new strategies are clearly required to target individual MMPs while limiting potential off-target effects. In this study, we describe the use of novel “bone-seeking” bisphosphonate based MMP-2 specific inhibitors for the treatment of myeloma *in vivo.* Our data show that BMMPIs can impact multiple myeloma tumor burden in addition to significantly protecting against the development of osteolytic lesions. We have also shown that MMP-2 can be specifically inhibited in the multiple myeloma-bone microenvironment underscoring the feasibility of selectively targeting MMPs in a tissue specific manner. We posit that this strategy could be adapted for the inhibition of other MMPs known to be important in skeletal malignancies thereby limiting potential toxicities associated with systemic MMP inhibition. Further refinement of the BMMPIs could lead to efficacious treatments for multiple myeloma. Given that bisphosphonates are well tolerated in humans, we anticipate that BMMPIs could be rapidly translated to the clinical setting.

## MATERIALS AND METHODS

All reagents were obtained from Fisher Scientific unless otherwise stated. Paraffin embedded deidentified multiple myeloma patient bone biopsies (*n* = 30) were obtained through the IRB approved Moffitt Total Cancer Care protocol (MCC50069). MMP expression in CD138+ cells was examined in the GEO series (Geo,http://www.ncbi.nlm.nih.gov/geo/) with the accession number GSE47552, performed by Lopez-Corall et al. [31]. This dataset contained gene expression analysis on 20 patients with monoclonal gammopathy of undetermined significance (MGUS), 33 with high-risk smoldering multiple myeloma (SMM) and 41 with multiple myeloma (MM). Five healthy donors were included in analysis, for comparison to normal conditions. The interaction between bone marrow MSCs, and multiple myeloma cells was investigated by Garcia-Gomez et al (GEO accession GSE46053) [32]. Briefly, MSCs isolated from healthy donors (*n* = 6) were co-cultured with the MM.1S human myeloma cell line for 24 hours using a transwell system. MSCs were isolated, and changes in gene expression were compared to MSCs cultured in isolation. Changes in MMP gene expression were determined using the NCIB GEO2R web application, making use of R-based statistical analysis. All animal experiments were performed under our University of South Florida approved Institutional Animal Care and Use Committee protocol (IS0000309). Luciferase-labeled Myeloma Cells (5TGM1-Luc) were obtained from Dr. Toshiyuki Yoneda through the University of Texas, Health Science Center at San Antonio, TX [47]. U266 luciferase expressing cells were obtained from Dr. Steven Grant at the University of Virginia, VA. OPM2 and MM.1S were obtained from the Deutsche Sammlung von Mikroorganismen und Zellkulturen (DSMZ) and American Tussue Culutre Collection (ATCC) respectively. These cells were cultured in RPMI containing 10% FBS, 1% penicillin and 1% streptomycin. All cell lines underwent periodic mycoplasma testing and were validated every 6 months *via* short tandem repeat (STR) profiling (IDEXX). The BMMPIs (ML104, ML111 and, ML115) were generated and characterized as previously described [26, 29].

### Immunofluorescent staining

Immunofluorescent procedures were conducted as previously described [19]. Briefly, paraffin-embedded sections (5μM) were rehydrated and blocked in serum followed by incubation overnight at 4°C with specific antibodies (MMP-2 #AB37150, Abcam, IgG2b #A90-109A-15, Bethyl Laboratories lnc, CD138 #B-A38, Kappa light chains #0198, Dako, Lambda Light chains #0199, Dako). Appropriate IgG controls were included for each of the antibodies being tested. After washing, species-specific flurophore conjugated secondary antibodies (Invitrogen) were used for the detection of primary antibodies. Slides were washed and then aqueously mounted with DAPI (4,6-Diamidino-2-phenylindole, dihydrochloride) containing media to visualize nuclei. All slides were imaged using an EVOS or Nikon fluorescent microscope at the Moffitt Analytical Microscopy Core.

### *In vitro* osteoclast differentiation assays

Whole bone marrow was flushed from the tibia of 6-8 week old male or female C57BL/6 recombinase activating gene-2 null (Rag2^−/−^) mice, and cultured in the presence of α-MEM and 25ng/ml macrophage colony stimulating factor (M-CSF#315-02, Peprotech) for 3 days. Non-adherent bone marrow cells were maintained in osteoclastogenic medium (100 ng/ml RANKL #47187000, OYC Americas and 20 ng/ml M-CSF) for 7 days. BMMPIs or Zoledronate (#SML0223, Sigma Aldrich) were added at varying concentrations (1 to 50μM) for 24 hours. Vehicle control wells were treated with 0.5% DMSO that reflected the final concentration of the vehicle in the 50μM drug treated groups. Subsequently, osteoclasts were fixed with 4% paraformaldehyde, and TRAcP stained (387A, Sigma-Aldrich) as previously described [48]. Multinucleated osteoclasts were counted using ImageJ.

### MTT assays

Myeloma cell lines were plated in 96-well plates at a density of 1×10^4^ cells/well. Cells were treated with vehicle or a range of BMMPI inhibitors or zoledronate (0.1 -100μM.) Cell viability was measured at 72 hours by the MTT assay following the manufacturer's instructions (CellTiter 96, #G3582, Pierce.) The absorbance was measured at 490nm after 4 hours of incubation at 37°C.

### Zymography

Conditioned media was collected from multiple myeloma and bone stromal cell lines following overnight culture in serum free media. Total protein was determined by the bicinchoninic acid (BCA) assay (Cat #23227, Pierce) according to manufacturer's instructions and protein concentration was normalized between samples prior to zymography. To determine MMP-2 enzymatic activity, gelatin was added to SDS resolving gels to a final concentration of 1 mg/ml and equal amounts of total protein (30μg) were run under non-reducing conditions. After electrophoresis, gels were washed in 2.5% solution of Triton-X-100 followed by overnight incubation in substrate buffer (50 mM Tris-HCL, pH 7.4 containing 5 mM CaCl_2_) at 37°C. The following day, the gels were stained (5 mg/ml comassie brilliant blue in 1:3:6 acetic acid: isopropanol: water) for 1 hour at room temperature prior to destaining in water and photographing using a standard gel imaging system. Recombinant MMP-2 (Cat # PF023, Calbiochem) was used a positive control for MMP-2.

### BMMPI treatment in mouse models of multiple myeloma

A total of 1 × 10^6^ murine 5TGM1 luciferase expressing cells were intravenously injected into 6-8 week old C57BL/KaLwRiji mice (*n* = 8). In separate experiments, 5 × 10^6^ human U266 luciferase expressing cells were injected into NOD-SCID-IL2rγ null (NSG) mice (*n* = 8). Tumor burden was monitored real-time using bioluminescence imaging (IVIS 200 system, Perkin Elmer) following intraperitoneal injection of 120mg/kg D-Luciferein (#LUCK-1G, Gold Biotechnology). Following detectable tumor burden in each model, mice were randomized and treated with Vehicle (PBS with 10% cyclodextran #AC29756, ACROS Organics), ML104 (1mg/kg), ML111 (1mg/kg), ML115 (1mg/kg) or zoledronate (100μg/kg) [49, 50]. U266 bearing mice were treated with vehicle, ML104 or zoledronate at the same dosage. Vehicle and treatment reagents were given by sub-cutaneous injection (100ul) three per times week. Clonal tumor expansion was validated using serum IgG2b (5TGM1) or IgE (U266) ELISA (Bethyl Laboratories, Cat# E90-109, E80-108). Mice were euthanatized upon reaching the clinical endpoint of hind limb paralysis, hunching or significant weight loss ( > 10%). Tumor bearing tibias were excised fixed in 10% buffered formalin overnight and stored in 70% ethanol. Subsequent to *ex vivo* imaging, bones were decalcified for 21 days in 14% EDTA at pH 7.4 at 4°C with changes every three days prior to processing and paraffin embedding.

### X-ray, microcomputed tomography (μCT), and histological analysis

Radiographic images (Faxitron X-ray Corp) were obtained using energy of 35kVp and an exposure time of 8 milliseconds. The spatial resolution is 10 lp/mm (48μm). The tumor volume (TuV) was calculated as a function of the total tissue volume (TV) of the tibial medullary canal using ImageJ software. For μCT analysis, the proximal tibia metaphyses were scanned (μCT-40; Scanco Medical). An evaluation of trabecular bone structural parameters was performed in a region that consisted of 1mm starting at 500μm from the growth plate. A three-dimensional cubical voxel model of bone was built and calculations were made for relative bone volume per total volume and trabecular number. Histological analysis was performed by staining sections with H&E, trichrome and tartrate resistant acid phosphatase (TRAcP) staining as previously described, and analyzed using ImageJ [48]. For histological analysis, the region of interest (ROI) for all treatment groups was identified by measuring 0.5mm from the growth plate, and analyzing the following 1mm area.

### MMP activity assays

At the study endpoint, supernatants from bone marrow flushes of subsets of tumor bearing limbs (*n* = 3) were collected from each group for *ex vivo* MMP enzymatic assays. Samples were kept at 4°C to avoid activation of latent MMPs. Prior to assaying for MMP activity, samples were normalized for total protein *via* BCA assay (#23225, Pierce). MMP-2 activity levels were measured (20μl/serum or bone marrow sample final volume) using an MMP-2 specific fluorigenic peptide according to manufacturer's instructions (#032014-01-384, EnSens). Fluorescence was read at Ex/Em 625-635 nm/ 655-665 nm (Victor^2^, PerkinElmer). Total MMP activity, was measured using a broad-spectrum fluorigenic substrate (OMNI-MMP, #BML-P126-0001, Enzo Life Sciences) according to the manufacturer's instructions. Samples were processed for MMP-2 activity using the assay described above. Fluorescence was read at Ex 320nm/Em 405nm for the OMNI-MMP probe.

### Statistical analysis

Statistical analyses were performed using GraphPad Prism (GraphPad Software, Inc).

## Abbreviations

BMMPI: bone-seeking matrix metalloproteinase inhibitors
ECM: extracellular matrix
MSC: mescenchymal bone stromal cell
MMP: Matrix Metalloproteinase
MMPI: matrix metalloproteinase inhibitor
MMP-2: matrix metalloproteinase-2
MMP: matrix metalloproteinase-9
Rag2^−/−^: recombinase activating gene-2
RANKL: Receptor activator of nuclear factor kappa-B ligand
TRA_c_P: Tartrate resistant acid phosphatase.

## References

[1] Shain KH, Dalton WS, Tao J (2015). The tumor microenvironment shapes hallmarks of mature B-cell malignancies. Oncogene.

[2] Mahindra A, Hideshima T, Anderson KC (2010). Multiple myeloma: biology of the disease. Blood reviews.

[3] Mahindra A, Laubach J, Raje N, Munshi N, Richardson PG, Anderson K (2012). Latest advances and current challenges in the treatment of multiple myeloma. Nature reviews Clinical oncology.

[4] Meads MB, Hazlehurst LA, Dalton WS (2008). The bone marrow microenvironment as a tumor sanctuary and contributor to drug resistance. Clin Cancer Res.

[5] Shay G, Hazlehurst L, Lynch CC (2016). Dissecting the multiple myeloma-bone microenvironment reveals new therapeutic opportunities. J Mol Med (Berl).

[6] Rogers MJ, Crockett JC, Coxon FP, Monkkonen J (2011). Biochemical and molecular mechanisms of action of bisphosphonates. Bone.

[7] Juarez P, Guise TA (2010). TGF-beta in cancer and bone: implications for treatment of bone metastases. Bone.

[8] Morgan GJ, Child JA, Gregory WM, Szubert AJ, Cocks K, Bell SE, Navarro-Coy N, Drayson MT, Owen RG, Feyler S, Ashcroft AJ, Ross FM, Byrne J (2011). Effects of zoledronic acid versus clodronic acid on skeletal morbidity in patients with newly diagnosed multiple myeloma (MRC Myeloma IX): secondary outcomes from a randomised controlled trial. The lancet oncology.

[9] Lipton A, Fizazi K, Stopeck AT, Henry DH, Brown JE, Yardley DA, Richardson GE, Siena S, Maroto P, Clemens M, Bilynskyy B, Charu V, Beuzeboc P (2012). Superiority of denosumab to zoledronic acid for prevention of skeletal-related events: a combined analysis of 3 pivotal, randomised, phase 3 trials. European Journal of Cancer.

[10] Lynch CC (2011). Matrix metalloproteinases as master regulators of the vicious cycle of bone metastasis. Bone.

[11] Van Valckenborgh E, Croucher PI, De Raeve H, Carron C, De Leenheer E, Blacher S, Devy L, Noel A, De Bruyne E, Asosingh K, Van Riet I, Van Camp B, Vanderkerken K (2004). Multifunctional role of matrix metalloproteinases in multiple myeloma: a study in the 5T2MM mouse model. Am J Pathol.

[12] Zdzisinska B, Walter-Croneck A, Kandefer-Szerszen M (2008). Matrix metalloproteinases-1 and -2, and tissue inhibitor of metalloproteinase-2 production is abnormal in bone marrow stromal cells of multiple myeloma patients. Leukemia research.

[13] Fowler JA, Mundy GR, Lwin ST, Lynch CC, Edwards CM (2009). A murine model of myeloma that allows genetic manipulation of the host microenvironment. Dis Model Mech.

[14] Bolkun L, Lemancewicz D, Sobolewski K, Mantur M, Semeniuk J, Kulczynska A, Kloczko J, Dzieciol J (2012). The evaluation of angiogenesis and matrix metalloproteinase-2 secretion in bone marrow of multiple myeloma patients before and after the treatment. Advances in medical sciences.

[15] Fu J, Li S, Feng R, Ma H, Sabeh F, Roodman GD, Wang J, Robinson S, Guo XE, Lund T, Normolle D, Mapara MY, Weiss SJ (2016). Multiple myeloma-derived MMP-13 mediates osteoclast fusogenesis and osteolytic disease. J Clin Invest.

[16] Barille S, Akhoundi C, Collette M, Mellerin MP, Rapp MJ, Harousseau JL, Bataille R, Amiot M (1997). Metalloproteinases in multiple myeloma: production of matrix metalloproteinase-9 (MMP-9), activation of proMMP-2, and induction of MMP-1 by myeloma cells. Blood.

[17] Barille S, Collette M, Thabard W, Bleunven C, Bataille R, Amiot M (2000). Soluble IL-6R alpha upregulated IL-6, MMP-1 and MMP-2 secretion in bone marrow stromal cells. Cytokine.

[18] Parmo-Cabanas M, Molina-Ortiz I, Matias-Roman S, Garcia-Bernal D, Carvajal-Vergara X, Valle I, Pandiella A, Arroyo AG, Teixido J (2006). Role of metalloproteinases MMP-9 and MT1-MMP in CXCL12-promoted myeloma cell invasion across basement membranes. J Pathol.

[19] Thiolloy S, Edwards JR, Fingleton B, Rifkin DB, Matrisian LM, Lynch CC (2012). An osteoblast-derived proteinase controls tumor cell survival via TGF-beta activation in the bone microenvironment. PLoS One.

[20] Coussens LM, Fingleton B, Matrisian LM (2002). Matrix metalloproteinase inhibitors and cancer: trials and tribulations. Science.

[21] Shay G, Lynch CC, Fingleton B (2015). Moving targets: Emerging roles for MMPs in cancer progression and metastasis. Matrix Biol.

[22] Fingleton B (2007). Matrix metalloproteinases as valid clinical targets. Curr Pharm Des.

[23] Teronen O, Laitinen M, Salo T, Hanemaaijer R, Heikkila P, Konttinen YT, Sorsa T (2000). Inhibition of matrix metalloproteinases by bisphosphonates may in part explain their effects in the treatment of multiple myeloma. Blood.

[24] Roelofs AJ, Coxon FP, Ebetino FH, Lundy MW, Henneman ZJ, Nancollas GH, Sun S, Blazewska KM, Bala JL, Kashemirov BA, Khalid AB, McKenna CE, Rogers MJ (2010). Fluorescent risedronate analogues reveal bisphosphonate uptake by bone marrow monocytes and localization around osteocytes in vivo. J Bone Miner Res.

[25] Roelofs AJ, Stewart CA, Sun S, Blazewska KM, Kashemirov BA, McKenna CE, Russell RG, Rogers MJ, Lundy MW, Ebetino FH, Coxon FP (2012). Influence of bone affinity on the skeletal distribution of fluorescently labeled bisphosphonates in vivo. J Bone Miner Res.

[26] Rubino MT, Agamennone M, Campestre C, Campiglia P, Cremasco V, Faccio R, Laghezza A, Loiodice F, Maggi D, Panza E, Rossello A, Tortorella P (2011). Biphenyl sulfonylamino methyl bisphosphonic acids as inhibitors of matrix metalloproteinases and bone resorption. ChemMedChem.

[27] Tauro M, Laghezza A, Loiodice F, Agamennone M, Campestre C, Tortorella P (2013). Arylamino methylene bisphosphonate derivatives as bone seeking matrix metalloproteinase inhibitors. Bioorganic & medicinal chemistry.

[28] Tauro M, Shay G, Sansil SS, Laghezza A, Tortorella P, Neuger AM, Soliman H, Lynch CC (2017). Bone-Seeking Matrix Metalloproteinase-2 Inhibitors Prevent Bone Metastatic Breast Cancer Growth. Mol Cancer Ther.

[29] Tauro M, Loiodice F, Ceruso M, Supuran CT, Tortorella P (2014). Dual carbonic anhydrase/matrix metalloproteinase inhibitors incorporating bisphosphonic acid moieties targeting bone tumors. Bioorganic & medicinal chemistry letters.

[30] Campestre C, Agamennone M, Tauro M, Tortorella P (2014). Phosphonate Emerging Zinc Binding Group in Matrix Metalloproteinase Inhibitors. Current Drug Targets.

[31] Lopez-Corral L, Corchete LA, Sarasquete ME, Mateos MV, Garcia-Sanz R, Ferminan E, Lahuerta JJ, Blade J, Oriol A, Teruel AI, Martino ML, Hernandez J, Hernandez-Rivas JM (2014). Transcriptome analysis reveals molecular profiles associated with evolving steps of monoclonal gammopathies. Haematologica.

[32] Garcia-Gomez A, De Las Rivas J, Ocio EM, Diaz-Rodriguez E, Montero JC, Martin M, Blanco JF, Sanchez-Guijo FM, Pandiella A, San Miguel JF, Garayoa M (2014). Transcriptomic profile induced in bone marrow mesenchymal stromal cells after interaction with multiple myeloma cells: implications in myeloma progression and myeloma bone disease. Oncotarget.

[33] Tauro M, Loiodice F, Ceruso M, Supuran CT, Tortorella P (2014). Arylamino bisphosphonates: potent and selective inhibitors of the tumor-associated carbonic anhydrase XII. Bioorganic & medicinal chemistry letters.

[34] Overall CM, Lopez-Otin C (2002). Strategies for MMP inhibition in cancer: innovations for the post-trial era. Nat Rev Cancer.

[35] Tauro M, Shay G, Sansil SS, Laghezza A, Tortorella P, Neuger AM, Soliman H, Lynch CC (2016). Bone seeking matrix metalloproteinase-2 inhibitors prevent bone metastatic breast cancer growth. Mol Cancer Ther.

[36] Oyajobi BO, Mundy GR (2003). Receptor activator of NF-kappaB ligand, macrophage inflammatory protein-1alpha, and the proteasome: novel therapeutic targets in myeloma. Cancer.

[37] Cook LM, Shay G, Aruajo A, Lynch CC (2014). Integrating new discoveries into the “vicious cycle” paradigm of prostate to bone metastases. Cancer Metastasis Rev.

[38] Lawson MA, Paton-Hough JM, Evans HR, Walker RE, Harris W, Ratnabalan D, Snowden JA, Chantry AD (2015). NOD/SCID-GAMMA mice are an ideal strain to assess the efficacy of therapeutic agents used in the treatment of myeloma bone disease. PLoS One.

[39] Slany A, Haudek-Prinz V, Meshcheryakova A, Bileck A, Lamm W, Zielinski C, Gerner C, Drach J (2014). Extracellular matrix remodeling by bone marrow fibroblast-like cells correlates with disease progression in multiple myeloma. Journal of proteome research.

[40] Nagase H, Woessner JF (1999). Matrix metalloproteinases. Journal Biological Chemistry.

[41] Sparano JA, Bernardo P, Stephenson P, Gradishar WJ, Ingle JN, Zucker S, Davidson NE (2004). Randomized phase III trial of marimastat versus placebo in patients with metastatic breast cancer who have responding or stable disease after first-line chemotherapy: Eastern Cooperative Oncology Group trial E2196. J Clin Oncol.

[42] Krzeski P, Buckland-Wright C, Balint G, Cline GA, Stoner K, Lyon R, Beary J, Aronstein WS, Spector TD (2007). Development of musculoskeletal toxicity without clear benefit after administration of PG-116800, a matrix metalloproteinase inhibitor, to patients with knee osteoarthritis: a randomized, 12-month, double-blind, placebo-controlled study. Arthritis research & therapy.

[43] Purushothaman A, Chen L, Yang Y, Sanderson RD (2008). Heparanase stimulation of protease expression implicates it as a master regulator of the aggressive tumor phenotype in myeloma. J Biol Chem.

[44] Kaushal GP, Xiong X, Athota AB, Rozypal TL, Sanderson RD, Kelly T (1999). Syndecan-1 expression suppresses the level of myeloma matrix metalloproteinase-9. Br J Haematol.

[45] Anderson KC (2015). Multiple myeloma: new uses for available agents, excitement for the future. J Natl Compr Canc Netw.

[46] Hu W, Zhang W, Li F, Guo F, Chen A (2014). Bortezomib prevents the expression of MMP-13 and the degradation of collagen type 2 in human chondrocytes. Biochem Biophys Res Commun.

[47] Mori Y, Shimizu N, Dallas M, Niewolna M, Story B, Williams PJ, Mundy GR, Yoneda T (2004). Anti-alpha4 integrin antibody suppresses the development of multiple myeloma and associated osteoclastic osteolysis. Blood.

[48] Cook LM, Araujo A, Pow-Sang JM, Budzevich MM, Basanta D, Lynch CC (2016). Predictive computational modeling to define effective treatment strategies for bone metastatic prostate cancer. Scientific reports.

[49] Croucher PI, De Hendrik R, Perry MJ, Hijzen A, Shipman CM, Lippitt J, Green J, Van Marck E, Van Camp B, Vanderkerken K (2003). Zoledronic acid treatment of 5T2MM-bearing mice inhibits the development of myeloma bone disease: evidence for decreased osteolysis, tumor burden and angiogenesis, and increased survival. J Bone Miner Res.

[50] Ory B, Heymann MF, Kamijo A, Gouin F, Heymann D, Redini F (2005). Zoledronic acid suppresses lung metastases and prolongs overall survival of osteosarcoma-bearing mice. Cancer.

